# Identification of long non-coding RNAs biomarkers for early diagnosis of myocardial infarction from the dysregulated coding-non-coding co-expression network

**DOI:** 10.18632/oncotarget.11999

**Published:** 2016-09-13

**Authors:** Chaoyu Sun, Hao Jiang, Zhiguo Sun, Yifang Gui, Hongyuan Xia

**Affiliations:** ^1^ Department of Cardiology, The Fourth Affiliated Hospital of Harbin Medical University, Harbin 150001, China; ^2^ Department of General Surgery, The Affiliated Hongqi Hospital of Mudanjiang Medical University, Mudanjiang 157011, China; ^3^ The Clinical Laboratory, The Affiliated Hongqi Hospital of Mudanjiang Medical University, Mudanjiang 157011, China

**Keywords:** biomarkers, diagnosis, long non-coding RNAs, myocardial infarction

## Abstract

Long non-coding RNAs (lncRNAs) have recently been shown as novel promising diagnostic or prognostic biomarkers for various cancers. However, lncRNA expression patterns and their predictive value in early diagnosis of myocardial infarction (MI) have not been systematically investigated. In our study, we performed a comprehensive analysis of lncRNA expression profiles in MI and found altered lncRNA expression pattern in MI compared to healthy samples. We then constructed a lncRNA-mRNA dysregulation network (DLMCEN) by integrating aberrant lncRNAs, mRNAs and their co-dysregulation relationships, and found that some of mRNAs were previously reported to be involved in cardiovascular disease, suggesting the functional roles of dysregulated lncRNAs in the pathogenesis of MI. Therefore, using support vector machine (SVM) and leave one out cross-validation (LOOCV), we developed a 9-lncRNA signature (termed 9LncSigAMI) from the discovery cohort which could distinguish MI patients from healthy samples with accuracy of 95.96%, sensitivity of 93.88% and specificity of 98%, and validated its predictive power in early diagnosis of MI in another completely independent cohort. Functional analysis demonstrated that these nine lncRNA biomarkers in the 9LncSigAMI may be involved in myocardial innate immune and inflammatory response, and their deregulation may lead to the dysfunction of the inflammatory and immune system contributing to MI recurrence. With prospective validation, the 9LncSigAMI identified by our work will provide additional diagnostic information beyond other known clinical parameters, and increase the understanding of the molecular mechanism underlying the pathogenesis of MI.

## INTRODUCTION

Myocardial Infarction (MI), commonly known as heart attack, is a serious result of coronary artery disease (CAD) caused by sudden blockage or extremely reduced blood flow in a coronary artery. MI remains the major cause of death and mortality globally, including China [1]. Over the past years, there is a sharply increasing trend in the morbidity of MI in China. Although statistics in 2011 suggested that there were about two million cases of MI accounting for 0.87% of cardiovascular disease (CVD)[2], it is estimated by that the number of patients with MI will increase to 23 million by 2030 [3]. Early diagnosis identifying subpopulations at high risk of having an infarct is crucial for deciding early tailored treatment to reduce MI mortality. Although currently available biomarkers, such as cardiac troponin and creatine kinase-MB (CK-MB), have used to assist with timely diagnosis [4, 5], some novel molecular biomarkers have highlighted their promising potentials and important roles for early management in MI.

Recent genome sequencing and transcriptomics analyses have revealed that only less than two percent of the human genome consists of protein-coding RNA, whereas the majority of the genome can be transcribed into RNA transcripts without protein coding capacity [6]. These non-coding RNAs (ncRNAs) can be classified into two types based on their size: short RNAs and long non-coding RNAs (lncRNAs). Short RNAs, including microRNAs (miRNAs), have been widely studied during the past ten years. Many studies have reported the dysregulation of miRNA expression in MI, including *miR-15* [7], *miR-21* [8], *miR-24* [9], *miR-29* [10] and so on. LncRNAs, a major class of ncRNAs, was defined as ncRNA transcript with greater than 200 nucleotides. Since the lncRNAs *H19* and *Xist* were firstly found to be involved in epigenetic regulation in the early 1990s [11, 12], increasing evidence has shown that lncRNAs are involved in the complex gene regulation network by as key molecular players at epigenetic, transcriptional and post-transcriptional levels [13, 14]. A handful of studies have revealed dysregulated expression of lncRNAs in a variety of disease states highlighting their potential clinical application as diagnostic and prognostic biomarkers or therapeutic targets in the pathology of diverse diseases, including cancers [15–17]. Some studies have reported several lncRNA-focus signatures which could be useful to predict patients' prognosis or metastasis in various cancers [18–28]. Recent studies have shown the close association between MI and altered lncRNA expression [29, 30], highlighting the potential of lncRNAs as biomarkers in early diagnosis of MI. However, lncRNA expression patterns and their predictive value for MI have not been systematically investigated.

In this study, we obtained lncRNA expression profiles and investigated the expression patterns between MI patients and healthy samples by re-annotating the publicly available Affymetrix microarray. Furthermore, we constructed and analyzed a dysregulated lncRNA-mRNA co-expression network that is associated with the occurrence of MI. We aimed to detect potential lncRNA biomarkers closely correlated with MI, and to develop novel lncRNA signature for identifying subpopulations at high risk of having an infarct

## RESULTS

### Identification of deregulated mRNA and lncRNA in AMI patients

We first performed SAM analysis to investigate the expression patterns of mRNAs and lncRNAs between AMI patients and healthy samples from the discovery cohort and identified 191 and 11 differentially expressed mRNAs and lncRNAs (logFC ≤1 or logFC >1, FDR-adjusted p <0.05). Among them, 175 mRNAs and 9 lncRNAs were over-expressed and 16 mRNAs and 2 lncRNAs were down-expressed in AMI patients compared with healthy samples (Supplementary Table S1).

### Construction and analysis of dysregulated lncRNA-mRNA co-expression network

**Figure 1:**
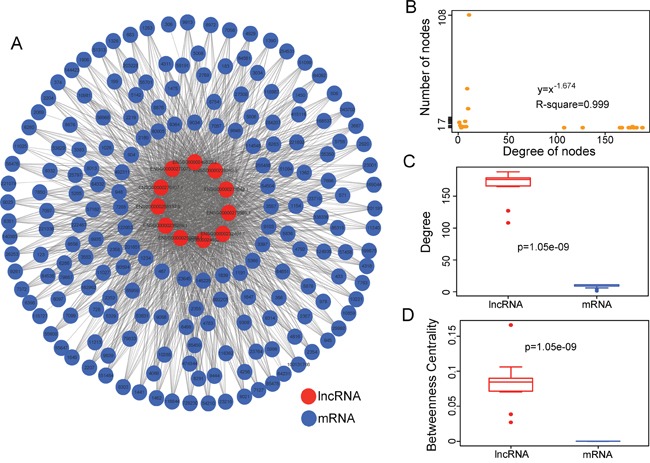
Construction and analysis of MI-related dysregulated lncRNA-mRNA co-expression network **A.** The DLMCEN generated by the procedure described in the Methods, including 1822 edges between 188 mRNAs and 11 lncRNAs. **B.** Degree distribution of the DLMCEN. **C.** The LncRNAs have significantly higher degrees than mRNAs in the DLMCEN. **D.** The lncRNAs have significantly higher betweenness centrality than mRNAs the DLMCEN.

We first investigated the co-expression correlations between differentially expressed mRNAs and differentially expressed lncRNAs. The lncRNA-mRNA pairs with a high PCC (>0.5) were selected as significantly dysregulated lncRNA-mRNA co-expression pairs and were integrated into the DLMCEN, in which there are 1822 edges between 188 mRNAs and 11 lncRNAs (Figure 1A). The investigation of the degree distribution of nodes in DLMCEN revealed a power-law distribution with a slope of −1.674 and R^2^ = 0.999 (Figure 1B), suggesting that DLMCEN has scale-free characteristics like many other biological networks distinguished from random networks. Moreover, the degree and betweenness centrality of lncRNAs are significantly higher than those of mRNAs (Wilcoxon rank-sum test) (Figure 1C and 1D), demonstrating that dysregulated lncRNAs tended to be hub nodes and played more important roles than mRNAs in the occurrence of AMI.

### Construction of SVM-based lncRNA signature in AMI diagnosis from the discovery cohort

**Figure 2:**
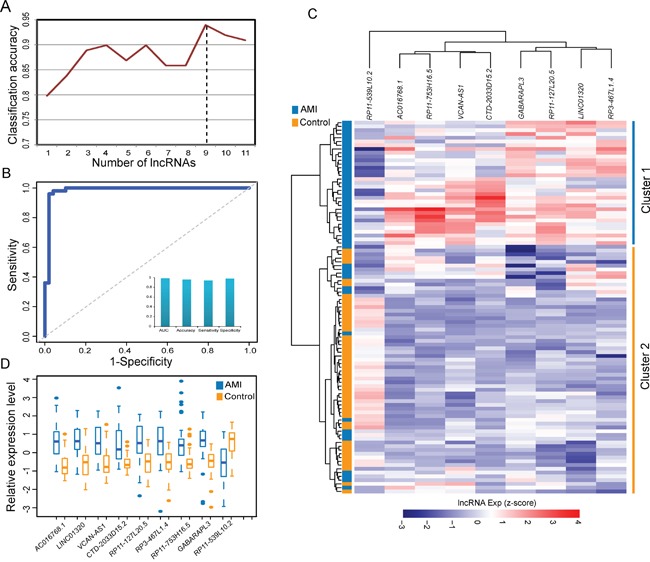
Identification and predictive value of SVM-based lncRNA signature in AMI diagnosis from the discovery cohort **A.** Classification performance of different numbers of lncRNA biomarkers. **B.** Performance evaluation of the 9LncSigAMI in early diagnosis using LOOCV procedure in the discovery cohort. **C.** The hierarchical clustering heat map of 99 samples based on expression profiles of 9 lncRNAs in the 9LncSigAMI in the discovery dataset. **D.** The expression levels of 9 lncRNAs in the 9LncSigAMI between MI patients and healthy samples

To identify an optimal lncRNA signature predictive of AMI, we have searched for lncRNA combinations among the 11 dysregulated lncRNAs in the DLMCEN, whose expression pattern can best distinguish AMI patients from the control samples, using SVM and LOOCV strategy in the discovery cohort. As shown in Figure 2A, a signature of 9 lncRNAs (termed 9LncSigAMI) with the highest accuracy was identified (Table 1). The classification of 99 samples in the discovery cohort using the 9LncSigAMI classifier achieved an accuracy of 95.96% with a sensitivity of 93.88% and a specificity of 98% (Figure 2B). The discriminatory power measured by AUC is 0.985 (Figure 2B). We also applied hierarchical clustering analysis to expression data of nine lncRNAs in the 9LncSigAMI from 49 AMI patients and 50 healthy samples and found 2 major sample clusters with clear differences in lncRNA expression patterns. As shown in Figure 2C, all healthy samples were grouped into Cluster 2 and most of AMI patients (33/49, 67.35%) were grouped into Cluster 1, revealing a significant association between lncRNA expression pattern and samples' disease status (p=5.431e-12, Chi-square test; Figure 2C). The above results demonstrated the good performance of the 9LncSigAMI in distinguishing AMI patients from healthy samples in the discovery cohort. Of these nine diagnostic biomarkers, eight lncRNAs tended to be risky lncRNAs whose up-regulated expression associated with AMI occurrence and only one lncRNAs was protective lncRNAs whose down-regulated expression associated with AMI occurrence (Figure 2C and 2D).

**Table 1:** The detailed information of lncRNA biomarkers in the 9LncSigAMI

LncRNA ID	Ensembl name	Genome locations	logFC
ENSG00000246526	RP11-539L10.2	Chr 4: 6,687,448-6,690,519 (−)	−1.02
ENSG00000232451	AC016768.1	Chr 2: 23,018,125-23,199,056 (−)	1.30
ENSG00000258086	RP11-753H16.5	Chr 12: 54,353,792-54,466,985 (+)	1.08
ENSG00000249835	VCAN-AS1	Chr 5: 83,531,352-83,581,320 (−)	1.26
ENSG00000276107	CTD-2033D15.2	Chr 15: 39,586,561-39,587,293 (+)	1.15
ENSG00000279980	GABARAPL3	Chr 15: 90,347,587-90,349,437 (+)	1.04
ENSG00000270075	RP11-127L20.5	Chr 10: 104,312,141-104,313,881(+)	1.11
ENSG00000228262	LINC01320	Chr 2: 34,677,555-34,738,231 (+)	1.27
ENSG00000236266	RP3-467L1.4	Chr 1: 7,810,242-7,827,342 (−)	1.08

### Validation of the 9LncSigAMI with an additional independent cohort

**Figure 3:**
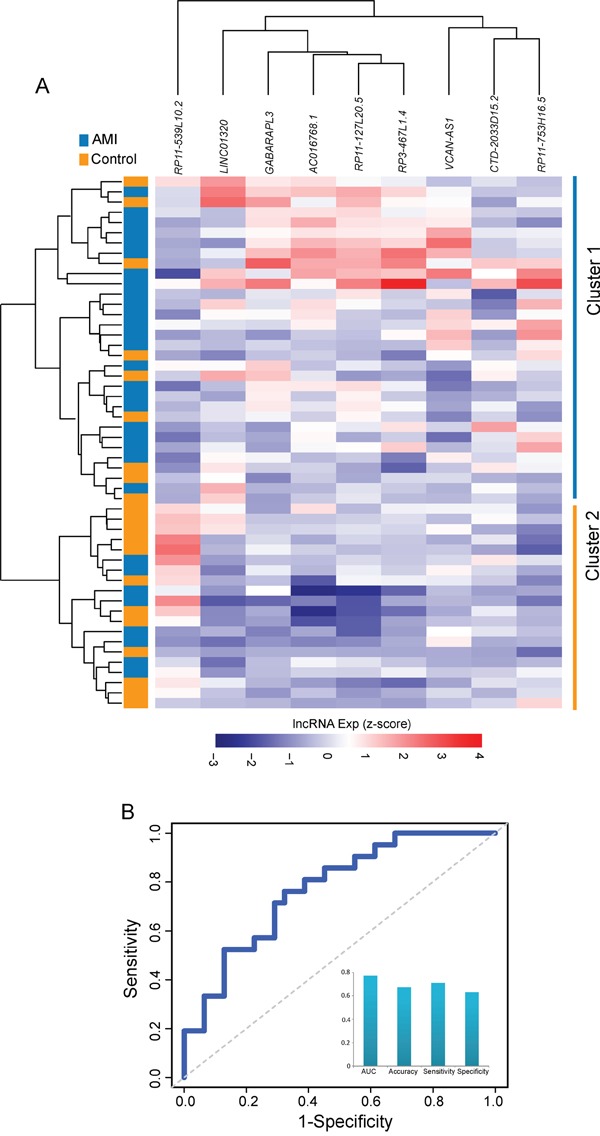
Validation of the 9LncSigAMI in the additional independent cohort **A.** The hierarchical clustering heat map of 52 samples based on expression profiles of 9 lncRNAs in the 9LncSigAMI in the test cohort. **B.** Performance evaluation of the 9LncSigAMI in early diagnosis using LOOCV procedure in the test cohort.

To evaluate the robustness of the 9LncSigAMI, we conducted a further validation of the predictive power of 9LncSigAMI using an additional independent cohort of 52 samples from Suresh's study [31] (denoted “validation cohort”). We first performed a hierarchical clustering analysis based on the expression pattern of these nine diagnostic biomarkers. 52 samples in the validation cohort were clearly clustered into two distinct subgroups (Figure 3A), with significantly different disease status (p= 2.27e-02, Chi-square test). As observed in the discovery cohort, eight protective lncRNAs showed a higher expression in healthy samples in Cluster 1 and one risky lncRNA showed a higher expression in AMI samples in Cluster 2.

We further assessed the predictive power of the 9LncSigAMI using SVM algorithm and LOOCV procedure. On the validation cohort, the 9LncSigAMI for distinguishing AMI patients from healthy samples achieves an AUC of 0.771 with a sensitivity of 70.97% and a specificity of 61.91% (Figure 3B). The 9LncSigAMI correctly classified 22 out of 31 AMI samples and 13 out of 21 control samples, resulting in 67.31% prediction accuracy. These results suggested that the 9LncSigAMI signature identified here was accurate and reliable for discriminating AMI patients from normal samples.

### Identification of associated biological pathways of the 9LncSigAMI

**Figure 4:**
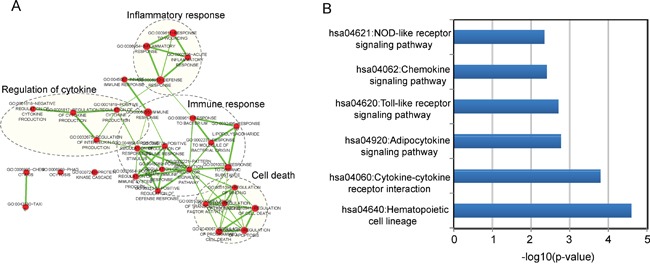
Function enrichment maps of the diagnostic lncRNAs **A.** The functional enrichment map of GO terms with each node represents a GO term and an edge represents the proportion of shared genes between connecting GO terms. **B.** The enriched KEGG pathways ranked by −log10 (p-value).

As an initial step to infer potential biological functions of the 9LncSigAMI, we first examined the expression correlation between mRNAs and nine lncRNAs using the Pearson correlation coefficient and identified 517 mRNAs positively correlated with at least one of the nine lncRNAs. Then GO and KEGG function enrichment analysis for mRNAs co-expressed with lncRNAs was conducted to identify associated biological processes and pathways of the 9LncSigAMI. Results with GO analysis revealed that mRNAs co-expressed with nine lncRNAs tended to be significantly enriched in 30 GO terms in the “Biological Process” (GOTERM-BP-FAT) (adjusted p-value < 0.10 and an enrichment score of >1.5) (Supplementary Table S2), which were grouped into four functional clusters including immune response, inflammatory response, regulation of cytokine and cell death (Figure 4A). The analysis of KEGG showed that mRNAs co-expressed with nine lncRNAs were involved in several pathways including Hematopoietic cell lineage, Cytokine-cytokine receptor interaction, Adipocytokine signaling pathway, Toll-like receptor signaling pathway, Chemokine signaling pathway and NOD-like receptor signaling pathway (Supplementary Table S3). These biological processes and pathways have been reported to be close with myocardial infarction [32–39], suggesting that the perturbation of nine lncRNAs in the 9LncSigAMI played important parts in the occurrence of AMI by interacting with mRNAs involved in known MI-related biological processes and pathways.

## DISCUSSION

Increasing evidence has revealed that lncRNA has complex and important roles in cardiovascular diseases, including MI [40, 41]. For example, the down-regulated expression of lncRNA *UCA1* has been observed at the early state of AMI patients [29]. Vausort and colleagues measured expression levels of five lncRNAs in 414 patients using quantitative PCR, and identified a lncRNA *MIAT* highly relevant to MI [30]. The above studies have demonstrated the potential roles of lncRNAs as promising novel biomarkers for the diagnosis and/or prognosis of MI. However, the current research strategies focused on molecular diagnostic or prognostic biomarkers have mainly analyzed expression profiles of mRNA or miRNAs [5] [42, 43]. Although transcriptome analysis has been reported in the AMI mouse model [44], there is a lack of the investigation into expression patterns and diagnostic values of lncRNA in MI patients owing to the limitation of available expression data in human. Recent studies revealed that lncRNA expression profiles could be obtained by re-annotating the probes in the commonly used microarrays [18, 19, 23, 45].

In this study, we obtained and analyzed lncRNA expression profiles of 151 samples (80 AMI patients and 71 healthy samples) by repurposing two publicly available microarray expression datasets to determine whether there is significantly altered lncRNA expression pattern between AMI patients and healthy samples. We observed significantly different lncRNA expression patterns in AMI patients compared to healthy samples and identified 11 differentially expressed lncRNAs, implying that these dysregulated lncRNAs may be associated with MI. Previous studies have demonstrated that lncRNAs function as key regulators of gene expression by interacting with protein-coding genes to participate in biological processes [46, 47]. Therefore, to identify functional lncRNAs and its partners, we investigated the co-expression relationship between dysregulated lncRNAs and dysregulated mRNAs and constructed a dysregulated lncRNA-mRNA co-expression network (DLMCEN). As general biological networks, the DLMCEN exhibited scale-free and modular characteristics. These dysregulated lncRNAs were found to be topologically central within DLMCEN and have maximal informational connections with other dysregulated mRNAs. After a comprehensive searching in both genetic association database (GAD) and Online Mendelian Inheritance in Man database (OMIM), 58 protein-coding genes in the DLMCEN have been found to be associated with cardiovascular disease, 18 of which were involved in MI (Supplementary Table S4). These observations indicated that the altered expression of these 11 lncRNAs in the DLMCEN contributed to the development of MI and could be used as potential biomarkers for early diagnosis of MI patients. Hierarchical clustering analysis revealed that these dysregulated lncRNAs in the DLMCEN were significantly correlated with disease status, highlighting their potential clinically application to assess the risk of MI and improve diagnosis prediction of MI. To identifying an optimal lncRNA signature considering a balance between accuracy and the number of lncRNAs, we used SVM and random forest method to detect a combination of lncRNAs that has a remarkable ability to distinguish AMI patients from healthy samples. After the feature selection procedure, 9 of 11 dysregulated lncRNAs was identified as optimal lncRNAs biomarkers with the highest accuracy. Thus, we developed an SVM-based diagnostic predictor as a lncRNA signature based on expression profiles of nine lncRNAs. The superior performance of the 9LncSigAMI in early AMI detection was further validated in the discovery cohort and another completely independent cohort.

Since only a few of lncRNAs have been annotated functionally, we performed bioinformatics analysis to infer possible associated biological roles of MI-related lncRNAs. From the co-expression network based on dysregulated lncRNAs and mRNAs, we performed functional enrichment analysis for co-expressed mRNAs and found that these mRNAs were enriched in several known MI-related biological processes and pathways. For example, TLR4, the members of the Toll-like receptors (TLR) family in the Toll-like receptor signaling pathway, have been found to play a detrimental role in myocardial ischemia/reperfusion (I/R) injury [48]. Cytokines, an inflammatory factor, were observed to be significantly up-regulated or down-regulated expression in AMI patients, and the altered cytokine expression had impacts on the immune functions in patients with AMI [33]. Chemokines, a family of chemotactic cytokines, acted as a modulator in complex biological processes such as cell proliferation and gene transcription, and its overexpression may be a reparative response following MI [37]. Therefore, it is a plausible inference that these nine lncRNA biomarkers may be involved in myocardial innate immune and inflammatory response, and their deregulation may lead to the dysfunction of the inflammatory and immune system contributing to MI recurrence.

In summary, we performed a comprehensive survey of the expression profiles of lncRNAs and coding RNAs from MI patients and healthy samples in a large of samples and constructed a dysregulated lncRNA-mRNA co-expression network which will improve our understanding of MI-related lncRNAs from a network view. Furthermore, we developed and validated a SVM-based lncRNA signature in use for early diagnosis of AMI with high accuracy. To our knowledge, this study is the first to access the application of lncRNAs for diagnostic prediction of MI. With prospective validation, the lncRNA signature identified by our work will provide additional diagnostic information beyond other known clinical parameters, and increase the understanding of the molecular mechanism underlying the pathogenesis of MI.

## MATERIALS AND METHODS

### Gene expression profile dataset

The gene expression profile data based on Affymetrix Human Genome U133 Plus 2.0 Array (HG-U133_Plus_2.0) from two independent nonoverlapping cohorts of AMI patients were obtained from the publicly available GEO database (www.ncbi.nlm.nih.gov/geo/). The initial discovery cohort of 49 AMI patients and 50 healthy samples were obtained from GEO (GSE66360, http://www.ncbi.nlm.nih.gov/geo/query/acc.cgi?acc=GSE66360) and used to identify novel lncRNAs biomarkers for AMI diagnosis. Another AMI patient dataset was also downloaded from GEO (GSE48060, http://www.ncbi.nlm.nih.gov/geo/query/acc.cgi?acc=GSE48060)[31], denoted “validation cohort”, including 31 AMI patients and 21 healthy samples and was considered as an independent test cohort to validate the diagnostic power of lncRNA biomarkers.

### Acquisition of lncRNA expression profiles

The raw expression profile data (.CEL files) of 99 samples in the discovery cohort and 52 samples in the validation cohort were downloaded from the GEO database. The probe set sequences of Affymetrix HG-U133_Plus_2.0 were obtained from the Affymetrix website (http://www.affymetrix.com). LncRNA expression data of 151 samples were obtained using the probe re-annotation strategy as previously described [18, 19, 24]. Briefly, probe sets of HG-U133_Plus_2.0 array were aligned to the human genome (GRCh38) and lncRNA gene sequence from GENCODE (release 23) using SeqMap tool with no mismatch [49]. Then lncRNA-specific probes were obtained by mapping the genomic locations of probes to the genomic locations of lncRNAs. Finally, expression data of 2332 lncRNA were obtained for further analysis.

### Expression profiles analysis

The significance analysis of microarrays (SAM) method was used to identify differentially expressed lncRNAs and mRNAs between AMI patients and healthy samples. The expression variation from AMI patients to healthy samples was characterized by logFC (log 2 fold change) and associated p-values adjusted after Benjamini-Hochberg false discovery rate (FDR) control approach [50]. Down- and up-regulated mRNAs and lncRNAs were selected with the cut-off criterion of a logFC < −1 and logFC >1 respectively, with FDR-adjusted p < 0.05. Hierarchical clustering analysis was carried out to investigate the patterns of lncRNA expression in the different samples, and the chi-square test was used to analyze the correlations between AMI status and lncRNA biomarkers.

### Construction and analysis of dysregulated lncRNA-mRNA co-expression network

The dysregulated lncRNA-mRNA co-expression network (DLMCEN) in AMI patients was constructed as follows: Firstly, Pearson correlation coefficient (PCC) was calculated by measuring the expression relationships between differentially expressed mRNAs and differentially expressed lncRNAs. Then lncRNA-mRNA pairs with a high PCC (>0.5) were selected as dysregulated lncRNA-mRNA co-expression pairs. Finally, a DLMCEN was constructed for AMI by assembling all dysregulated lncRNA-mRNA co-expression pairs identified above. A node represents a lncRNA or mRNA, and mRNA and lncRNA are connected if they are differentially co-expressed.

### Construction of SVM-based lncRNA signature in AMI diagnosis

A lncRNA-focus predictive signature for sample classification was developed using the support vector machine (SVM) with the sigmoid kernel. The performance of SVM-based lncRNA signature was estimated using the leave one out cross-validation (LOOCV). Sensitivity, specificity and accuracy were calculated through a 2 × 2 contingency table, and the ROC curve was drawn by plotting true positive rates (sensitivity) against false positive rates (1-specificity).

To construct an optimal lncRNA signature in AMI diagnosis, optimal lncRNA biomarkers were selected using the random forest supervised classification algorithm as follows: (i) candidate lncRNA biomarkers were ranked according to their random forest importance value. (ii) The SVM-based signature was developed by adding one lncRNA at a time in a top-down order starting with the first two lncRNAs in the lncRNA ranking list, and the performance of the SVM-based signature was evaluated using LOOCV. (iii) The optimal number of lncRNA biomarkers in the signature could be found when achieving the highest classification accuracy.

### Functional analysis of lncRNA biomarkers

The expression correlations between lncRNA biomarkers and mRNAs were calculated using the Pearson correlation coefficients (PCCs). The mRNAs positively correlated with biomarkers (PCCs>0.60) was chosen as co-expressed mRNAs associated with lncRNA signature. We performed bioinformatics analysis to predict the function of lncRNA signature by functional enrichment analysis of Gene Ontology (GO) and Kyoto encyclopedia of genes and genomes (KEGG) for co-expressed mRNAs. Functional enrichment analysis was carried out using DAVID Bioinformatics Tool (version 6.7) which is widely used to discover the biological implications of a set of genes [51]. Enriched GO terms limited in “Biological Process” (GOTERM-BP-FAT) and KEGG pathways with an adjusted p-value of <0.10 using the Benjamini-Hochberg procedure and an enrichment score of >1.5 were considered as significant functional annotations. *Enrichment maps of* significant GO terms were constructed and visualized using the Enrichment Map plugin in Cytoscape software [52].

## SUPPLEMENTARY MATERIALS TABLES










