# Supplementary material for: Identification of long non-coding RNAs biomarkers for early diagnosis of myocardial infarction from the dysregulated coding-non-coding co-expression network
Source: Oncotarget. 2016 Sep 13;7(45):73541–51. doi: 10.18632/oncotarget.11999 (PMC5341997; doi:10.18632/oncotarget.11999)
Supplement: Supplementary file 1 [file oncotarget-07-73541-s001.pdf]

# Identification of long non-coding RNAs biomarkers for early diagnosis of myocardial infarction from the dysregulated coding-non-coding co-expression network

## SUPPLEMENTARY TABLES

**Supplementary Table S1: Differentially expressed mRNAs and lncRNAs between AMI patients and healthy samples from the discovery cohort**

See Supplementary File 1

**Supplementary Table S2: Significantly enriched GO terms**

See Supplementary File 2

**Supplementary Table S3: Significantly enriched KEGG pathways**

See Supplementary File 3

**Supplementary Table S4: Detailed information of protein-coding genes involved in MI in the DLMCEN**

See Supplementary File 4
